# Comparison of cerebral cortex activation induced by tactile stimulation between natural teeth and implants

**DOI:** 10.4317/jced.57463

**Published:** 2020-11-01

**Authors:** Daiki Sekido, Takero Otsuka, Tateshi Shimazaki, Akinori Ohno, Kei Fuchigami, Koudai Nagata, Tetsutaro Yamaguchi, Katsuhiko Kimoto

**Affiliations:** 1 Department of Oral Interdisciplinary Medicine, Division of Orthodontic, Graduate School of Dentistry, Kanagawa Dental University, Kanagawa, Japan; 2 Department of Oral Interdisciplinary Medicine, Division of Prosthodontics and Oral Implantology, Graduate School of Dentistry, Kanagawa Dental University, Kanagawa, Japan

## Abstract

**Background:**

The purpose of this study was to assess the cortical-level sensory differences between natural teeth with a periodontal membrane and dental implants.

**Material and Methods:**

We used functional near-infrared spectroscopy (fNIRS) to measure brain activity in the cerebral cortex of 12 patients who had both natural teeth and dental implants in the lower molar region. Painless vibratory tactile stimulation was performed on both the natural teeth and the dental implants.

**Results:**

Activation was seen in the somatosensory cortex during stimulation of both natural teeth and dental implants. A comparison of cortical activation showed no significant differences between natural teeth and dental implants.

**Conclusions:**

These results indicate the possible existence of sensory input to the cerebral cortex via dental implants as well as natural teeth, and thus suggest that may not only the periodontal membrane be involved in the signaling pathway. The data from this experiment may help us for understanding the neural mechanisms underlying natural teeth and dental implants.

** Key words:**fNIRS, natural teeth, implants, brain activity, somatosensory cortex.

## Introduction

Many recent studies have addressed the association between oral function and brain activity, and according to Shimazaki *et al.*, the areas of brain activity evoked by painless vibrotactile stimuli of natural teeth vary according to the type of tooth (1). It is believed that sensory input from the periodontal membrane not only induces sensory area activity but also affects masticatory motor function in the form of feedback (2). Meanwhile, the effects of tooth loss and deterioration of oral function on brain activity reportedly go beyond loss of coordination of masticatory movement to also influence cognitive function (3). Studies have also found that occlusal disharmony causes the production of stress-responsive plasma corticosterone, resulting in decreased bone density as a secondary effect (4,5). In light of these reports, there are concerns that the effects of tooth loss are not limited to occlusal dysfunction but also have a systemic impact.

Although prosthetic treatment options for tooth loss have conventionally included bridge prosthodontics and partial or complete dentures, dental implant treatment has come to be widely used in clinical dentistry in recent years (6). Dental implant treatment restores oral function, with masticatory function closer to that of natural teeth in comparison with other protheses (7,8). However, comparisons of the sensitivity thresholds of dental implants and natural teeth that have investigated sensations, including pressure, touch, and vibration, on the basis of self-reported subjective data (9-11) have found that natural teeth can distinguish significantly less intense stimuli, and have described differences in perception depending on the presence or absence of the periodontal membrane, and the importance of this membrane in occlusal sensation. Nevertheless, other studies have reported that even patients with full-mouth dental implants can distinguish the hardness of test materials (12), and according to Kimoto *et al.*, brain activity during mastication with implant overdentures similarly that during normal mastication (13), suggesting that some sensory input is provided even by implants lacking a periodontal membrane.

Objective methods of measuring brain activity include positron emission tomography (PET), magnetoencephalography (MEG), functional magnetic resonance imaging (fMRI), and functional near-infrared spectroscopy (fNIRS). PET, MEG, and fMRI all provide high spatial resolution but require a shield room for measurements, restricting the position of the subject during scanning (14). fNIRS is a noninvasive technique that uses near-infrared light to measure cerebral blood flow, and since the device is small, it permits the subjects to vary their position and move during measurements (15).

In this study, we focused on the effect of the periodontal membrane and used fNIRS to compare the cortical activation induced by the stimulation of natural teeth and dental implants in the mouths of the same subjects.

## Material and Methods

-Subjects

Twelve volunteers (two men, ten women; mean age = 59 yrs; range, 38-71 yrs) who were being treated at Kanagawa Dental University Hospital participated in the study. According to the participants’ self-reports, they were all right-handed. None of the participants had a history of neurological or psychiatric illness, and all were free from active caries and periodontitis. All participants had full occlusal support with natural teeth or dental implants, and healthy periodontal tissue. The subjects selected were those with natural teeth and dental implants on the lower jaw, allowing data from natural teeth and dental implants to be measured from the same oral cavity. We obtained written informed consent from each participant. The study protocol was approved by the local ethics committee (approval number 505).

-Functional Near-infrared Spectroscopy

In this study, we used 48 ch fNIRS (ETG7100, Hitachi Medical Co., Kashiwa, Japan) to measure brain activity. The fNIRS has two types of probe and can be located in each of the left and right hemispheres. The probe groups contain eight illuminators and eight detectors, respectively. The near infrared light reflected was detected between illuminator and detector, and the change in cerebral blood flow was measured by the amount of reflection. This device measures oxyhemoglobin (oxy-Hb) and deoxyhemoglobin (deoxy-Hb) and their sum (total hemoglobin; total-Hb), in 0.1 s units using two kinds of wavelengths (695 ± 20 nm and 830 ± 20 nm). The distance between the illuminator and the detector is 30 mm, and it can receive infrared signals up to about 20 mm from the scalp surface. The depth of 20 mm from the scalp contained scalp blood flow in addition to cerebral blood flow. Among these changes in blood flow, scalp blood flow was excluded as noise by performing task-responsive principal component analysis. It has been reported that oxy-Hb was more effective than deoxy-Hb in assessing brain activity (16). Therefore, we used the change of oxy-Hb concentration as an index of brain activity. In this study, the position of the probe was set based on the international 10/20 method used in electroencephalography. The horizontal position was set so that the second column of the probe was parallel to the line (A1-Cz-A2) connecting the left and right auricular regions and passing through the vertex. The vertical position was set so that probe located on the second row from the top matched C3/C4. These probes covered the left and right somatosensory cortices. A 6DOF digitizer (Patriot, Polhemus, Colchester, VT, USA) was used to record relative position information between the anatomical reference point and each probe. Further, the spatial registration proposed by Singh *et al.* (17) was used to project the probe positions onto the Montreal Neurological Institute (MNI) standard brain space. The projected channel position was estimated by the software (nfri_mni_estimation; freeware available at http://www.jichi.ac.jp/brainlab/indexE.html) based on the report by Okamoto *et al.* (18) the channel corresponding to the sensory cortex was selected. In addition, the recorded cerebral blood flow data was projected on a brain map using NIRS-SPM software (Bio Imaging Signal Processing Lab, Daejeon, South Korea; freeware available from http://bisp.kaist.ac.kr/NIRS-SPM.html). Changes in cerebral blood flow corresponding to stimulation in each group were identified with an uncorrected level of *p* <0.005. (Ye *et al.*, (19)).

-Vibrotactile Stimulation Task 

**Figure 1:**
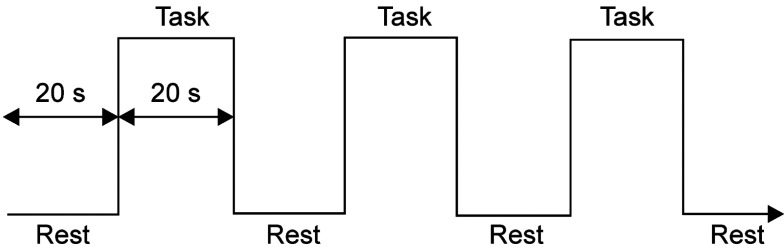
Schematic illustration of the task design.

All participants were stimulated by the natural tooth on either the left or right side of the lower jaw, and received the same stimulation on the contralateral dental implant. In this research, we used a motor (IMPLANTER Neo Plus, Kyocera Medical Co., Ltd., Osaka, Japan) to control the rotation speed and force so that the same stimulus could be applied, and a dental handpiece (EVA-ER4, NSK Nakanishi Japan, Tochigi, Japan) that moved in a reciprocating up and down motion to produce the same stimulus as tapping. The tip of the handpiece was covered with resin to prevent damage to the target natural teeth and dental implants. The handpiece was a contra-angle type and capable of applying stimulation in the same direction as the tooth axis. The stimulus interval was 500 min-1 and the applied force value was set to 5.0 N. All data measurements were performed by the same operator. Before recording the data, a short stimulation test was performed to confirm that the tip of the handpiece touched only touched the target natural teeth or dental implants and did not cause the pain associated with stimulation. The vibrotactile stimulation were given three times in a single sequence. There was a rest period before and after stimulation, and each duration was 20 seconds (Fig. 1). The order of applying stimuli was randomly assigned. During the recording of data, participants were instructed to remain stationary to avoid body movement artifacts. To avoid temporal muscle movement artifacts, participants bit a bite block made of silicon so as not to interfere with the stimulus, so that the mouth opening remained unchanged during data recording. The above tasks were performed in a quiet private room that did not stimulate sight, hearing, or smell.

-Data Analysis (hemoglobin data) 

In the present study, the amount of change in oxy-Hb in response to stimulation was evaluated as cerebral cortex activity. Of the stimulation tasks given to each natural teeth and dental implants, the amount of oxy-Hb increased in response to the three periods of stimulation was averaged to obtain oxy-Hb data for each group. Brain regions that responded to stimuli were identified by the probabilistic estimation method proposed by Singh *et al.* (17) and NIRS-SPM software (Bio Imaging Signal Processing Lab; Ye *et al.*, (19)). It was identified that the left and right probe channels 9 corresponded to the somatosensory cortex as the sites where the activity was observed. A comparison between the two groups was performed using the average value of oxy-Hb data obtained when the natural teeth and the dental implants were stimulated. Student’s t-test was used for this comparison.

## Results

**Figure 2:**
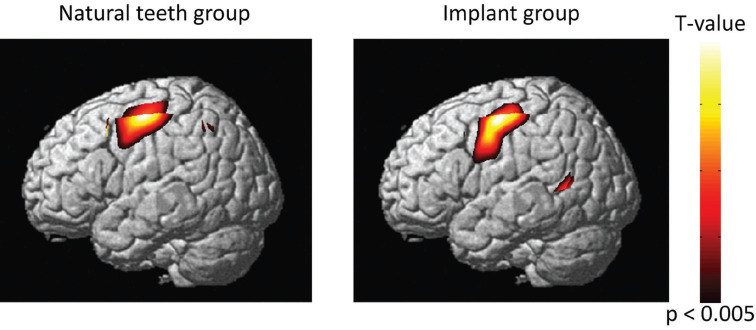
Brain activation maps were created from group analysis of each vibrotactile stimulation task (12 participants; *P* < 0.005, uncorrected). The t value is indicated by the color code.

**Figure 3:**
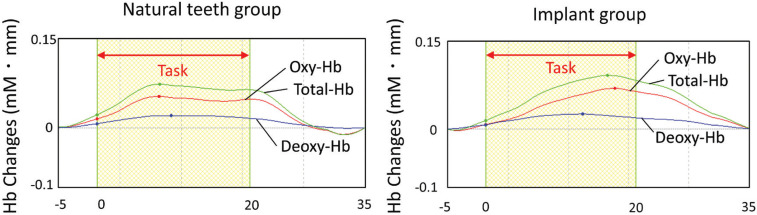
The typical time-course changes in hemoglobin in the primary somatosensory cortex. Task-related changes in oxy-Hb (red), deoxy-Hb (blue), and total-Hb (green) levels. The red arrow represents the time of the vibrotactile stimulation task.

**Figure 4:**
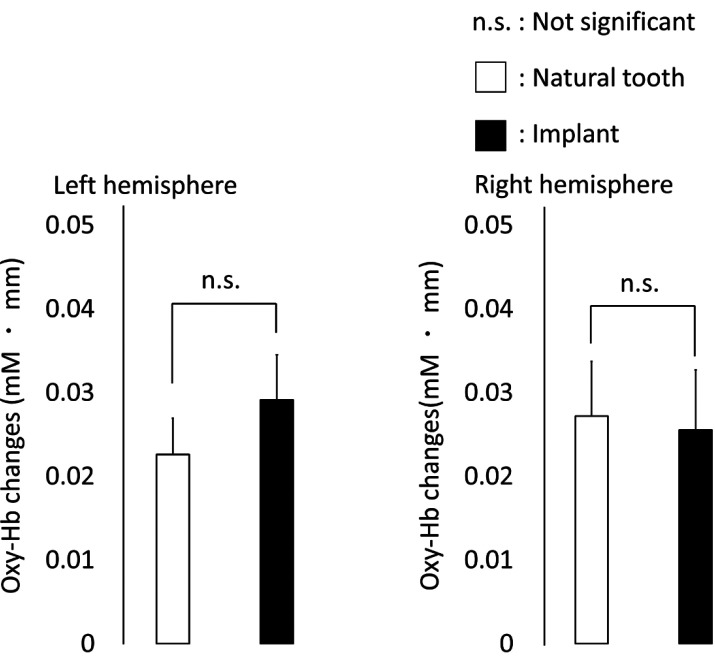
Changes in oxy-Hb causing by each vibrotactile stimulation. Comparison between the natural teeth group and implant group in the left and right primary somatosensory cortices. In both sides, no significant difference was seen in the increase in oxy-Hb levels. Error bars indicate SE. (* Montreal Neurological Institute (MNI)).

The probe positions measured by the 6DOF digitizer corresponded to channel 9 in the somatosensory cortex on the left and right (Table 1). The location of the probe was identified by a probabilistic estimation method (17). The activation maps of the 12 participants’ output by NIRS-SPM showed activation of the somatosensory cortex in both the dental implant group and natural teeth group (Fig. 2). In the somatosensory cortex, we found increased levels of oxy-Hb and total-Hb during vibrotactile stimulation both the natural teeth and dental implants groups (Fig. 3). The maximum value appeared about 10 seconds after the start of the task, and then slowly returned to the baseline Oxy-Hb changes in the somatosensory cortex of natural teeth and dental implants were not significantly different in the two groups using Student’s t-test (*p*> 0.05) (Fig. 4). In addition, there was no significant difference in the amount of oxy-Hb during the stimulation between participants with the dental implant on the right side and those with the dental implant on the left side. This result was the same for natural teeth. In comparison of the oxy-Hb mean levels in the somatosensory cortex, there was no significant difference between left and right hemispheres.

**Table 1:**
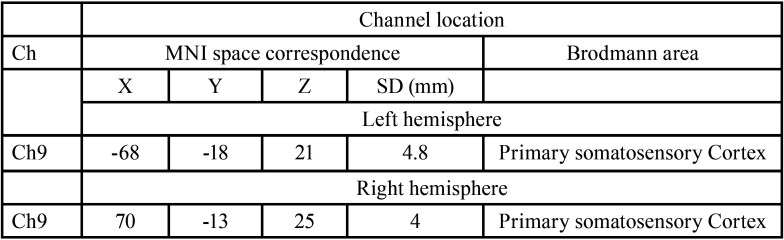
Channel location areas. This table shows the brain regions which is estimated from MNI co-ordinates.

## Discussion

Our results confirmed that vibrotactile stimulation to dental implants lacking a periodontal membrane evoked activity in the primary somatosensory cortex of the cerebrum corresponding to the oral cavity in the same way as for natural teeth.

The mechanoreceptors in the periodontal membrane are reportedly so sensitive that they can sense changes equivalent to the thickness of a hair (19). However, it has been reported that even dental implants lacking such periodontal membrane perceive the sensation of chewing something (11,20). Although some studies comparing sensation in natural teeth and dental implants have concluded that natural teeth have significantly greater sensibility (21), those studies compared the sensory threshold, and the stimuli applied to the teeth were less intense than the forces generated during mastication (8-11). In this study, in accordance with Shimazaki *et al.*’s report of the association between vibrotactile stimulation of natural teeth and the primary somatosensory cortex (1), we observed similar changes in cerebral blood flow when comparatively intense vibrotactile stimulation corresponding to mastication was applied to both natural teeth and dental implants.

The frequency of the vibrotactile stimulus was set to induce primary somatosensory cortex activation when this stimulus was applied to the lower molar region, in accordance with the studies by Tamura *et al.* (22) and Shimazaki *et al.* (1). The observed sites of primary somatosensory cortex activation due to vibrotactile stimuli at this frequency were consistent with those previously reported, suggesting that the use of this frequency was valid. The results of NIRS-SPM channel location analysis and group analysis identified the site of activity as the primary somatosensory cortex, a result consistent with the report by Shimazaki *et al.* Similar changes over time in oxy-Hb levels during vibrotactile stimulation were observed in both the dental implants group and the natural teeth group, and this waveform was also the same as that reported by previous studies.

According to Shimazaki *et al.*, the area activated by vibrotactile stimulation of natural teeth is significantly larger for molars than for incisors, canines, or premolars, and they found no difference between left and right or upper and lower molars (1). We only used lower teeth and dental implants at the molar region in this study, and found no bilateral difference in brain activation regions. However, because we did not apply vibrotactile stimuli to upper or non-molar teeth in this study, further studies are necessary to investigate the effect of the dental implant insertion site on the activation region.

Vibrotactile stimuli are believed to be transmitted by the mandible because of the direct integration of the implant with the jawbone, which is a characteristic of dental implants. Activation of the oral area of the primary sensory cortex by masticatory movements other than dental stimulation has also been reported (12), and the primary somatosensory cortex is also activated by input from mechanoreceptors in structures such as the temporomandibular ligament and the periodontal membranes of the adjacent natural teeth even if large movements are not involved, suggesting the transmission of compensatory afferent information to replace that from implants (23).

In conclusion, we confirmed that dental implants transmit afferent information to the cerebral cortex during vibrotactile stimuli and activate the primary somatosensory cortex in the same way as natural teeth, suggesting that may not only the periodontal membrane affect primary somatosensory cortex activation. However, the perioral region contains numerous tissues capable of sensory perception, and further studies under a wider range of conditions are required to identify the compensatory mechanisms responsible for these effects.
